# Single-Cell RNA Sequencing Reveals Potential Mechanism of RUNX3 Reshaping Tumor Microenvironment in Non-small-cell Lung Cancer

**DOI:** 10.1245/s10434-025-18034-w

**Published:** 2025-09-07

**Authors:** Weijin Xiao, Jianqing Zheng, Yujie Deng, Bifen Huang, Weibin Liu, Xuejin Zheng, Kaijia Zhou, Weimin Fang, Xiaohui Chen

**Affiliations:** 1 Department of Pathology, Clinical Oncology School of Fujian Medical University, Fujian Cancer Hospital, Fuzhou, China; 2 Department of Radiation Oncology, The Second Affiliated Hospital of Fujian Medical University, Quanzhou, China; 3 Department of Medical Oncology, The First Affiliated Hospital of Fujian Medical University, Fuzhou, China; 4 Department of Obstetrics and Gynecology, People’s Hospital Affiliated of Quanzhou Medical College, Quanzhou, China; 5 Department of Thoracic Surgery, Clinical Oncology School of Fujian Medical University, Fujian Cancer Hospital, Fuzhou, Fujian Province China; 6 Interdisciplinary Institute of Medical Engineering of Fuzhou University, Fuzhou, China; 7 Department of Neurosurgery, Clinical Oncology School of Fujian Medical University, Fujian Cancer Hospital, Fuzhou, China; 8 Department of Medical Oncology, Fuzhou Hospital of Chinese Medicine affiliated to Fujian University of Chinese Medicine, Fuzhou, China

**Keywords:** Non-small-cell lung cancer, RUNX3, Tumor-associated macrophage, Tumor microenvironment, Single-cell RNA sequencing

## Abstract

**Background:**

RUNX3 acts as a tumor suppressor gene in non-small-cell lung cancer (NSCLC), yet its specific biological mechanism is still unclear. This study aimed to uncover tumor microenvironment (TME) changes in NSCLC with varying RUNX3 expression statuses through single-cell RNA sequencing.

**Patients and Methods:**

In total, seven patients with NSCLC with detailed pathological data were involved, with three both paracancerous and cancerous tissue samples. After sequencing, the “Seurat” package was used to analyze differentially expressed genes, annotate cell clusters with marker genes, and compare cell proportion differences at different RUNX3 expression levels. Observed-over-expected cell number ratios (*R*_o/e_) assessed cell type enrichment among three pathological types.

**Results:**

Immunohistochemical staining of RUNX3 categorized three patients into the RUNX3-negative group (RUNX3_Neg) and four into the RUNX3 positive group (RUNX3_Pos). All cells were classified into 13 types based on marker genes. *R*_o/e_ results showed fibroblasts were the only enriched cell type in RUNX3_Pos cancer tissue, while club cells, ciliated cells, and so on were enriched in RUNX3_Neg cancer tissue. RUNX3_Neg tissues were more likely to accumulate certain immune cells compared with RUNX3_Pos tissues. *R*_o/e_ also indicated RUNX3_Neg cancer tissues were more prone to macrophage depletion, while RUNX3_Pos tissues were more prone to macrophage enrichment.

**Conclusions:**

Through single-cell sequencing, our study found that RUNX3 expression status is closely related to NSCLC TME. Mononuclear phagocytes may be an important target cell population for RUNX3 to change TME.

**Supplementary Information:**

The online version contains supplementary material available at 10.1245/s10434-025-18034-w.

Lung cancer (LC) still ranks the top mortality rate among all types of malignancies and poses the greatest threat to human health and life worldwide,^1^ demonstrating 2.481 million new cases every year and accounting for 12.4% of new cancer cases in the world.^2^ LC accounts for 22% of cancer-related deaths, far more than any other type of cancer,^3^ and non-small-cell lung cancer (NSCLC) is the most common type, accounting for 85% of LC.^4^ The majority of patients with LC are diagnosed at advanced stages and therefore demonstrate a poor prognosis.^5^ Targeted therapies targeting on sensitive oncogenic driver genes have greatly improved the clinical efficacy of locally advanced NSCLC, especially the prognosis and survival quality of advanced NSCLC have been significantly improved.^6^

The enhanced expression of oncogenes or the inhibitory expression of tumor suppressor genes is the most important molecular event in the occurrence and development of cancer.^7,8^ Previous studies have shown that the progression of cancer is precisely regulated by gene networks.^8,9^ Blocking these molecular events with drugs would probably retard tumor growth and/or even trigger tumor apoptosis.^10^ The successful practice of targeted therapy for NSCLC has proven that blocking lung cancer driver genes is a highly feasible treatment.^11^ Therefore, identifying the core target genes for targeted therapy is crucial and of great therapeutic value.

Runt-related transcription factors (RUNX) play an important role in a variety of cellular biological processes, such as cell cycle progression, differentiation, apoptosis, immunity, and epithelial mesenchymal transformation.^12^ There are three main members in the mammalian RUNX family, encoded by genes RUNX1, RUNX2, and RUNX3, and each of them has a different tissue expression profile, which means that the RUNX family has a very different function in mammals.^12,13^ Although present in different tissues, all RUNX genes have a major regulatory role and are associated with cancer.^12^ Paradoxically, the tumor suppressor or carcinogenic function of RUNX gene varies according to tumor type and stage, suggesting that the function of RUNX gene is extremely complex.^14^ More and more evidences show that RUNX3 has a tumor-suppressive role in various cancers, which means that RUNX3 is a tumor suppressor gene (TSG).^15–17^ Our previous study has revealed that loss of RUNX3 expression in NSCLC was correlated with shorter overall survival and worse prognosis.^18^ However, unlike RUNX1 and RUNX2, little is known about the role of RUNX3 in NSCLC, and its function has not been fully studied.^19^ In recent years, single-cell sequencing technology has developed rapidly, which can reveal changes in the tumor microenvironment (TME) at the cellular level, laying the foundation for exploring biological differences in different disease status. Here, in the present study, we aim to reveal the changes in cellular microenvironment in NSCLC under different RUNX3 expression statuses through single-cell sequencing.

## Patients and Methods

### Patients and Sample Collection

**Fig. 1:**
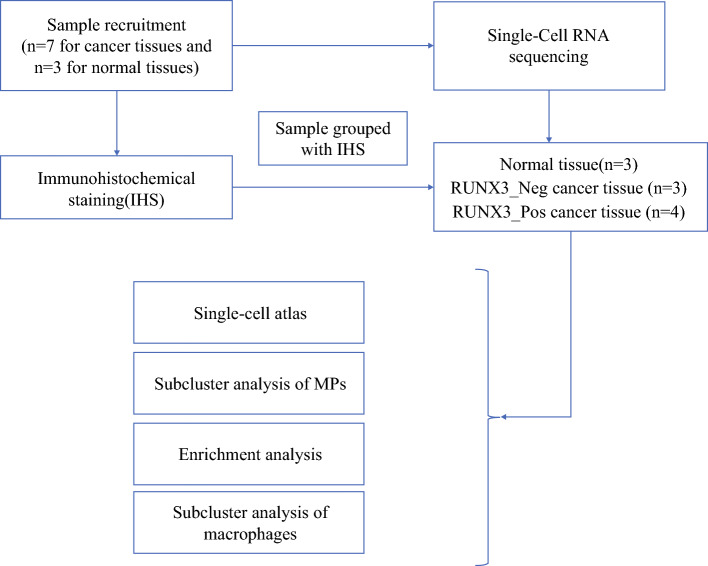
Flow chart of study design

A total of seven patients that were pathologically diagnosed with NSCLC from Fujian Medical University Cancer Hospital were investigated in this study, including five cases of adenocarcinoma and two cases of squamous cell carcinoma. Among them, two cases of adenocarcinoma and one case of squamous cell carcinoma simultaneously provided both cancerous tissue and adjacent tissue. Fresh tumor or adjacent normal lung tissue were surgically resected from the above-described patients (as shown in Supplementary Table S1). All subjects provided written informed consent, and this study was approved by the institutional ethics committee of Fujian Medical University Cancer Hospital (K2024-413-01). The flowchart of this study is shown in Fig. 1.

### Tissue Dissociation and Preparation of Single-Cell Suspensions

Within 30 min after surgery, fresh tissues from patients who met the inclusion criteria were stored in sCelLiveTM Tissue Preservation Solution (Singleron) on ice and washed three times with Hanks’ balanced salt solution (HBSS) prior to dissociation. The sCelLiveTM Tissue Dissociation Solution (Singleron) were used to lyse tissue samples, digest cells, and obtain single-cell suspensions. The tissue dissociation was finished by Singleron PythoN™ Tissue Dissociation System at 37 °C for 15 min. The GEXSCOPE^®^ red blood cell lysis buffer (RCLB, Singleron) was added to remove red blood cells. A dead cell removal kit (Miltenyi Biotech, 130-090-101) was used to remove inactive cancer cells, thereby improving cell viability to meet the requirements of single-cell RNA sequencing. Finally, the samples were stained with Trypan blue and the cell viability was evaluated microscopically.

### Single-Cell RNA Sequencing and Library Construction

After treatment with phosphate-buffered saline (PBS) (HyClone), single-cell suspensions with a cell density of 2 × 105 cells/mL were loaded onto a microwell chip using the Singleron Matrix^®^ Single Cell Processing System. Barcoding beads are subsequently collected from the microwell chip. Then, reverse transcription of the mRNA captured by the barcoding beads was conducted to obtain complementary DNA (cDNA) followed by PCR amplification. The amplified cDNA is then fragmented according to previously reported research protocols and then ligated with sequencing adapters.^20^ The single-call RNA sequencing (scRNA-seq) libraries were constructed according to the protocol of the GEXSCOPE^®^ Single-Cell RNA Library Kits (Singleron) on BD Rhapsody system and Singleron platform.^21^ Individual libraries were diluted to 4 nM, pooled, and sequenced on Illumina novaseq 6000 with 150-bp paired end reads. Quality control, batch correction, and major cell type annotation of single-cell RNA sequencing data were carried out according to the reported protocol.^22^

After obtaining read counts of single-cell gene expression, standard bioinformatics analysis procedures were used for data analysis. The content of bioinformatic analyses included differential gene expression analysis, gene enrichment analysis, single-cell trajectories analysis, and so on.

### Immunohistochemical Staining (IHS) of RUNX3 Protein in Human NSCLC Samples

IHC of RUNX3 protein was completed by the pathologists in our hospital. A primary mouse monoclonal antibody to RUNX3 (R3-5G4, 1:100, ab224641, Abcam) was used. Specimens obtained from surgically resected tissue were cut into 5-μm sections. Immunohistochemistry was performed using an automatic system, DAKO Autostainer Link48 (DAKO). Semiquantitative analysis of IHC staining was finished by two independent pathologists (W.X. and Y.D.) who were blinded to the included patients with NSCLC. The percentage of positively stained immunoreactive cells and the staining intensity were assessed to determine the expression of RUNX3. The percentage of positive cells was rated as follows: 0 points, < 10%; 1 point, 10–50%; and 2 points, > 50%. The staining intensity was rated as follows: 0 points (no staining or weak staining: light yellow), 1 point (moderate staining: yellow brown) and 2 points (strong staining: brown). The overall score of RUNX3 is the sum of the positive percentage and staining intensity, ranging from 0 to 4 points. For the next, the seven patients were divided into a RUNX3_Neg group (an overall score between 0 and 2) and a RUNX3_Pos group (an overall score between 3 and 4). Two pathologists resolved any differences in the overall score through discussion.

### Statistical Analysis

Statistical analyses were performed by using R 4.3.2. Student’s *t*-tests and one-way analysis of variance (ANOVA) tests were used to compare the statistical difference of the continuous variable. In this study, all statistical significance may be marked as: ns, not significant, *P < 0.05, **P < 0.01, or ***P < 0.001. We used the ratio of observed over expected cell numbers (*R*_o/e_) to explore the enrichment tendency of different cell types in three pathological types.^23^ In brief, the expected cell numbers of each cell subtype in each tissue were obtained from the chi-squared test, and *R*_o/e_ > 1 in a tissue indicated preference of this cell subtype in this tissue.

### Ethics Statement

This study was approved by the Fujian Cancer Hospital Ethics Committee. The study was conducted in accordance with the ethical standards of the institutional and/or national research committee and with the 1964 Helsinki Declaration and its later amendments or comparable ethical standards.

## Results

### IHS Results of RUNX Expression Status in the Samples Included

**Fig. 2:**
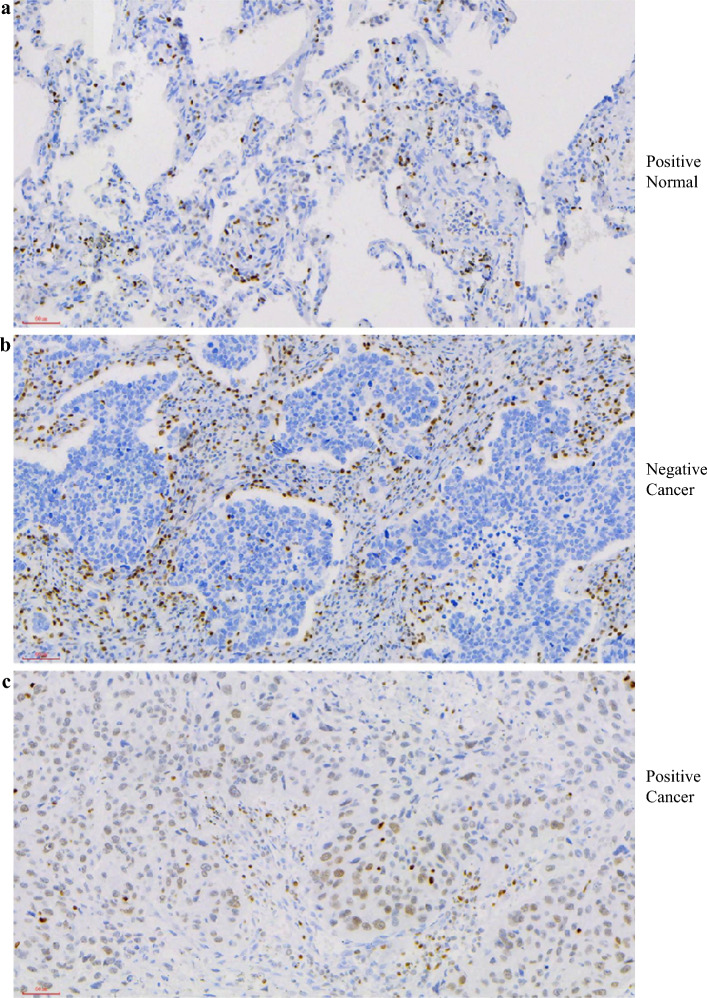
Interpretation of RUNX3 in immunohistochemical staining; **a** a case of a patient with RUNX3-positive expression in adjacent cancer tissues; **b** a case of a patient with RUNX3-negative expression in cancer tissues; **c** a case of a patient with RUNX3-positive expression in cancer tissues

In total, seven tumor tissues and three adjacent normal tissues from patients diagnosed with NSCLC were selected, and the IHS results of RUNX3 in seven patients with NSCLC are shown in Supplementary Table S1. According to IHS characteristics of RUNX3, three patients were divided into the RUNX3-negative group (RUNX3_Neg), and four patients were divided into RUNX3-positive group (RUNX3_Pos). The IHS characteristics of RUNX3 in several patients are shown in Fig. 2.

### Single-Cell Atlas Among Normal Tissue, RUNX3_Neg Cancer Tissue, and RUNX3_Pos Cancer Tissue

**Fig. 3:**
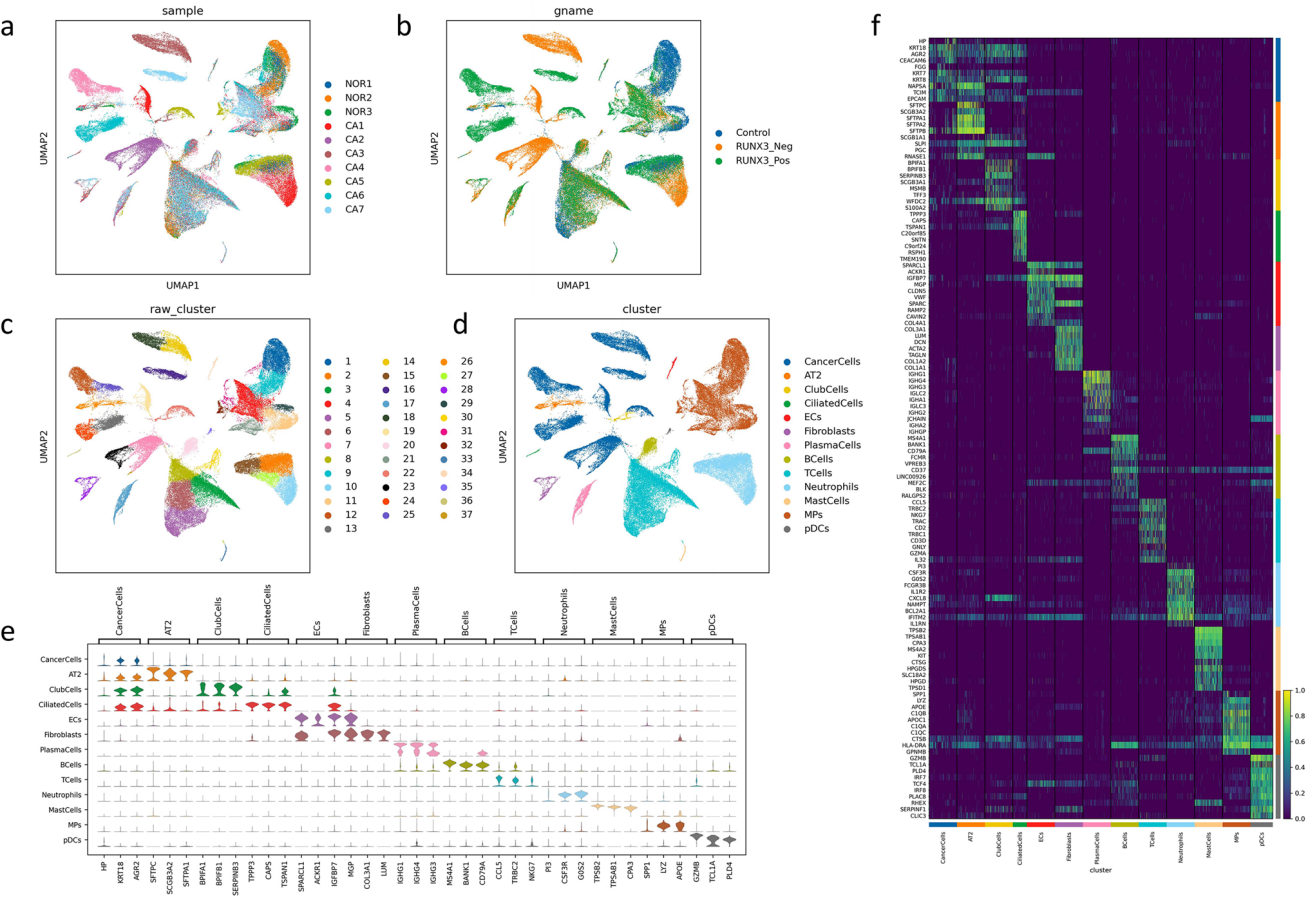
Single-cell atlas among normal tissue, RUNX3_Neg cancer tissue, and RUNX3_Pos cancer tissue; **a** single-cell atlas across all samples (*NOR* normal tissues, *CA* cancer tissues); **b** single-cell atlas among normal tissues (control), RUNX3_Neg cancer tissue, and RUNX3_Pos cancer tissue; **c** UMAP dimensionality reduction clustering graph using unsupervised clustering across all cancer tissues; **d** UMAP dimensionality reduction clustering graph using unsupervised clustering across all cancer tissues was used to divide cells into 13 categories; **e** the top 30 marker genes expressed in each cell type are shown in dot plot; **f** the marker genes expressed in each cell type are shown in heat map

The landscape and pathogenesis of TME between normal tissue and cancer tissue were analyzed via scRNA-seq analysis and results were shown in Fig. 3a. The cell subclusters were classified on the basis of histological type and RUNX3 expression status, as shown in Fig. 3b. This single-cell atlas contains a total of 95,997 cells. Unsupervised clustering was used for preliminary cell classification, and the cells can be classified into 37 types, as shown in Fig. 3c. Based on the expression of marker genes in different cells (as shown in Supplementary Table S2), these cells can be classified into 13 types, including cancer cells (CancerCells), alveolar type II cells (AT2), club cells (ClubCells), ciliated cells (CiliatedCells), endothelial cells (ECs), fibroblasts, plasma cells (PlasmaCells), B cells (BCells), T cells (TCells), neutrophils, mast cells (MastCells), mononuclear phagocytes (MPs), and plasma dendritic cells (pDCs), as shown in Fig. 3d. The marker genes expressed in each cell type are shown in Fig. 3e and f.

**Fig. 4:**
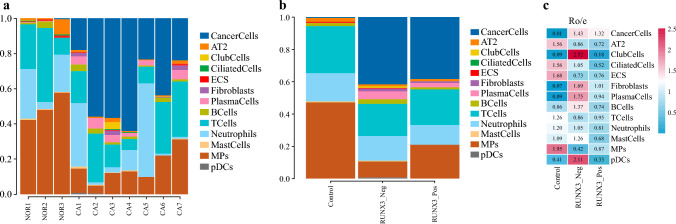
The percents of cell subclusters in each sample and group; **a** the percents of cell subclusters in each sample; **b** the percents of cell subclusters in each group; **c** the results of *R*_o/e_ of cell subclusters in each sample and group

The percents of cell subclusters in each sample and group were shown in Supplementary Table S3 and S4 and Fig. 4a and b. The results of *R*_o/e_ are shown in Supplementary Table S5 and Fig. 4c. From the results in Fig. 4c, it can be seen that the only cell type enriched in RUNX3_Pos cancer tissue was fibroblasts, while the cell types that enriched in RUNX3_Neg cancer tissue were club cells, ciliated cells, fibroblasts, plasma cells, B cells, neutrophils, mast cells, and pDCs. It can also be observed that compared with normal tissues, MPs exhibit a depleted status in both RUNX3_Pos cancer tissue and RUNX3_Neg cancer tissue, indicating that monocyte depletion is an important microenvironmental feature of NSCLC. From the perspective of immune cells, RUNX3_Neg tissues are more likely to accumulate plasma cells, B cells, mast cells, and plasmacytoid dendritic cells compared with RUNX3_Pos tissues.

### Subcluster Analysis of MPs Among Normal Tissue, RUNX3_Neg Cancer Tissue, and RUNX3_Pos Cancer Tissue

MPs are a general term for a class of myeloid immune cells that originate from hematopoietic stem cells and have phagocytic and/or antigen-presenting functions, including monocytes, macrophages, and dendritic cells. As mentioned prior, depletion of MPs is an important microenvironmental feature of NSCLC. We will further annotate MPs and classify them into subclusters. In our study, downregulation of MPs is an important characteristic of immune microenvironment change caused by loss of RUNX3 expression. Therefore, we focused on the changing characteristics of MPs and further subdivide the MPs cell cluster into different subclusters.

**Fig. 5:**
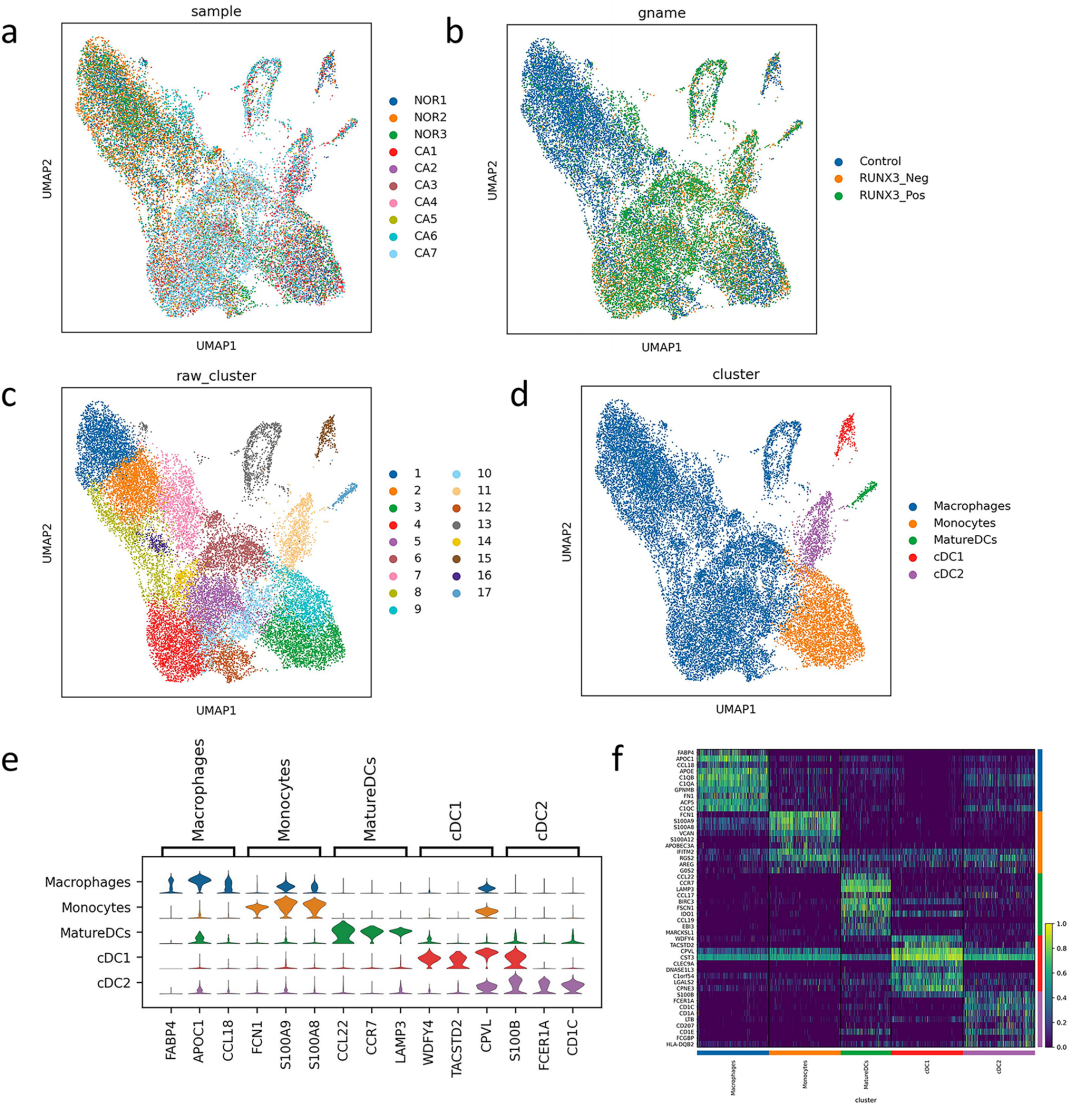
Single-cell atlas of mononuclear phagocytes (MPs) among normal tissue, RUNX3_Neg cancer tissue, and RUNX3_Pos cancer tissue; **a** single-cell atlas across all samples (*NOR* normal tissues, *CA* cancer tissues); **b** single-cell atlas among normal tissues (control), RUNX3_Neg cancer tissue, and RUNX3_Pos cancer tissue; **c** UMAP dimensionality reduction clustering graph using unsupervised clustering across all cancer tissues; **d** UMAP dimensionality reduction clustering graph using unsupervised clustering across all cancer tissues was used to divide cells into five categories; **e** the top marker genes expressed in each cell type are shown in dot plot; **f** the marker genes expressed in each cell type are shown in heat map

In our study, a total of 23,054 cells were identified as MPs, as shown in Fig. 5a and b. Unsupervised clustering showed that the cells can be classified into 17 types, as shown in Fig. 5c. Based on the marker genes in Supplementary Table S2, five different subtypes were identified through detailed annotation, including macrophages, monocytes, mature dendritic cells (MatureDCs), conventional type 1 dendritic cells (cDC1), and conventional type 2 dendritic cells (cDC2), as shown in Fig. 5d. The marker genes expressed in each cell are shown in Fig. 5e. According to the logarithmic values of folding changes, a heatmap was drawn for the top ten differentially expressed genes in different cell types, and the results are shown in Fig. 5f.

**Fig. 6:**
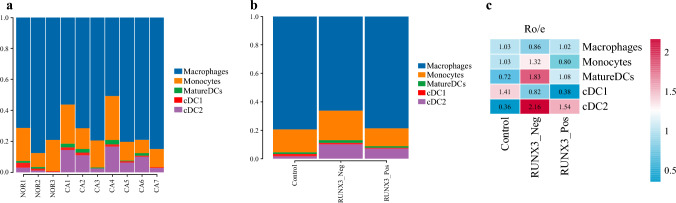
The percents of cell subclusters of mononuclear phagocytes (MPs) in each sample and group; **a** the percents of cell subclusters in each sample; **b** the percents of cell subclusters in each group; **c** the results of *R*_o/e_ of cell subclusters in each sample and group

The percents of subclusters from MPs in each sample and group are shown in Supplementary Table S6 and S7 and Fig. 6a and b. The results of *R*_o/e_ are shown in Supplementary Table S8 and Fig. 6c. From the results in Fig. 6c, it can be seen that RUNX3_Neg cancer tissues are more prone to macrophage depletion, while RUNX3_Pos tissues are more prone to macrophage enrichment. On the contrary, RUNX3_Neg cancer tissues are more prone to monocyte enrichment, while RUNX3_Pos tissues are more prone to monocyte depletion. Compared with normal tissues, both types of cancer tissues are prone to mature dendritic cell enrichment and cDC2 enrichment but are more prone to cDC1 depletion. Mature dendritic cell and cDC2 are more prone to be enriched in RUNX3_Neg cancer tissues. Our single-cell atlas revealed that the alteration of MPs may be important single-cell characteristics of RUNX3 gene status change in NSCLC.

### Single Cell Gene Set Enrichment Analysis

Gene differential expression analysis was performed in all MPs, and the differentially expressed genes (DEGs) are shown in Supplementary Table S9. Heatmap and volcano maps showing the expression level of some DEGs among normal tissue, RUNX3_Neg cancer tissue, and RUNX3_Pos cancer tissue are presented in Supplementary Fig. S1a–f. The gene set enrichment analysis was conducted in MPs subset with GO functional annotation and Kyoto Encyclopedia of Genes and Genomes (KEGG) pathways enrichment.

The results of the top ten pathways for each enrichment analysis are shown in Supplementary Fig. S2a–d (each large figure contains four small figures).

### Subcluster Analysis of Macrophages among Normal Tissue, RUNX3_Neg Cancer Tissue, and RUNX3_Pos Cancer Tissue

The results of *R*_o/e_ from Fig. 6c showed that macrophage depletion was found in RUNX3_Neg cancer tissues; therefore, subcluster analysis of macrophages among normal tissue, RUNX3_Neg cancer tissue, and RUNX3_Pos cancer tissue was conducted in this part. In our study, a list of marker genes including AZU1, CXCL10, FABP4, SELENOP, SPP1, and STMN1 was used for subcluster analysis of macrophages. Here, we define this subcluster analysis as the new six-gene classification.

**Fig. 7:**
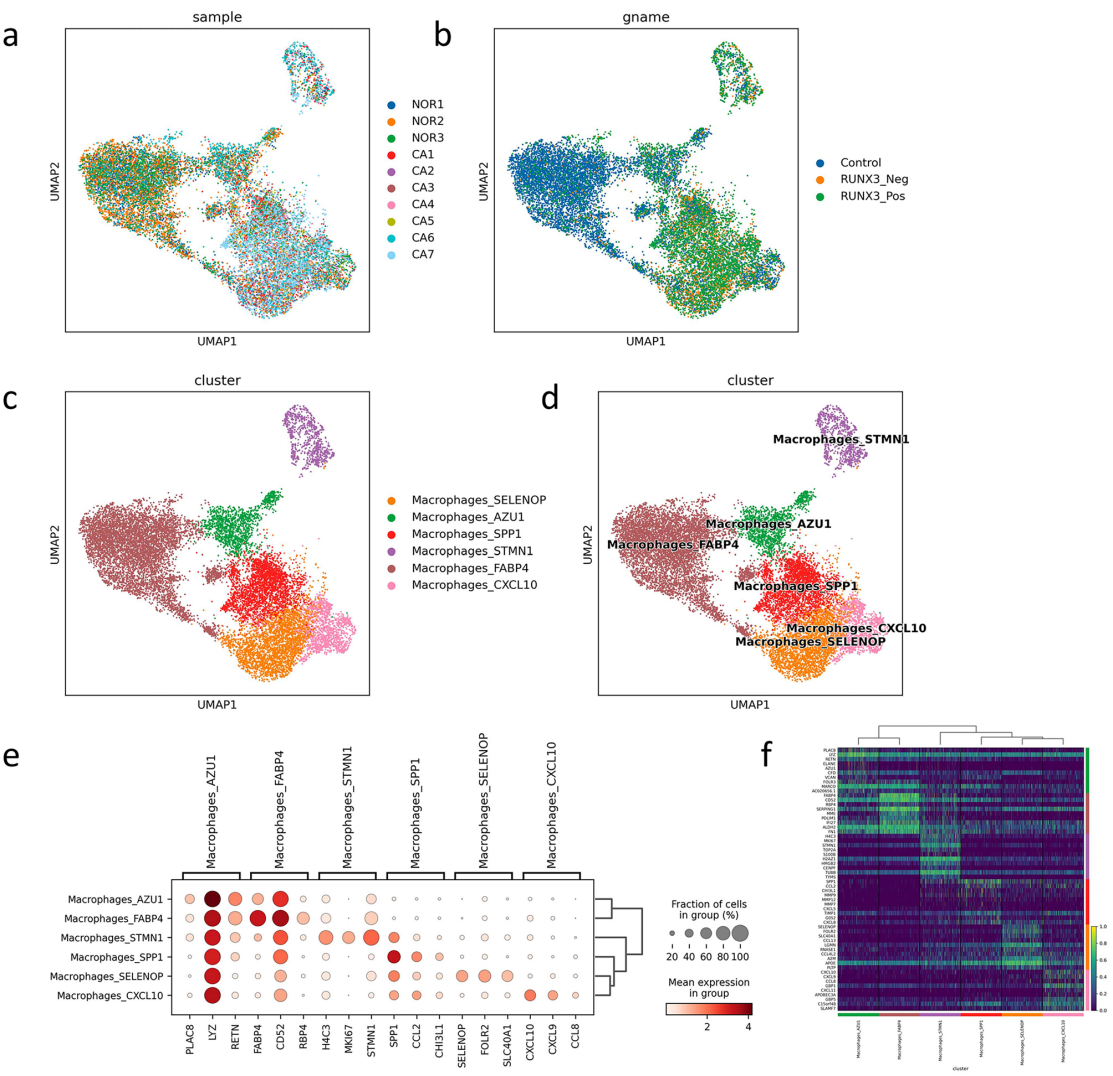
Single-cell atlas of macrophages among normal tissue, RUNX3_Neg cancer tissue, and RUNX3_Pos cancer tissue; **a** single-cell atlas across all samples (*NOR* normal tissues, *CA* cancer tissues); **b** single-cell atlas among normal tissues (control), RUNX3_Neg cancer tissue, and RUNX3_Pos cancer tissue; **c** UMAP dimensionality reduction clustering graph using unsupervised clustering across all cancer tissues; **d** UMAP dimensionality reduction clustering graph using unsupervised clustering across all cancer tissues was used to divide cells into five categories; **E** the top marker genes expressed in each cell type are shown in dot plot; **f** the marker genes expressed in each cell type are shown in heat map

Subcluster analysis of macrophages among normal tissue, RUNX3_Neg cancer tissue, and RUNX3_Pos cancer tissue is shown in Fig. 7a and b. Unsupervised clustering showed that the cells can be classified into six types, as shown in Fig. 7c. Based on the marker genes list, six different subtypes were identified through detailed annotation, including Macrophages_AZU1, Macrophages_CXCL10, Macrophages_FABP4, Macrophages_SELENOP, Macrophages_SPP1, and Macrophages_STMN1, as shown in Fig. 7d. The marker genes expressed in each cell were shown in Fig. 7e. According to the logarithmic values of folding changes, a heatmap was drawn for the top differentially expressed genes in different cell types, and the results are shown in Fig. 7f.

The percents of subclusters from macrophages in each sample and group were shown in Supplementary Fig. S3a and b. The results of *z*-scores are shown in Supplementary Fig. S3c. The results of *R*_o/e_ are shown in Supplementary Fig. S3d. The analysis results showed that Macrophages_FABP4 exhibited significant depletion in cancer tissues, while Macrophages_SLENOP, Macrophages_SPP1, Macrophages-STMN1, and Macrophages_CXCL10 showed significant enrichment. Macrophages_AZU1 is quite unique, exhibiting opposite infiltration characteristics in RUNX3_Pos and RUNX3_Neg tissues, with enrichment in RUNX3_Po tissues and depletion in RUNX3_Neg tissues, as shown in Supplementary Fig. S3e.

We further compared the M1 and M2 scores among three tissue types and macrophage subclusters. The distribution characteristics of macrophages included in the analysis are shown in Supplementary Fig. S4a and b (grouped by histological type). Except for Macrosphages_AZU1, the other five macrophage subpopulations showed different distribution differences of M1 scores in the three histological types, as shown in Supplementary Fig. S4c. From the perspective of macrophage subclusters, the M1 score of Macrophages_CXCL10 is the highest, while the score of Macrophages_STMN1 is the lowest, as shown in Supplementary Fig. S4d.

The similar results of M2 score were shown in Supplementary Fig. S5a–d. The results showed different distribution differences of M2 scores in the three histological types, as shown in Supplementary Fig. S5c. The M2 score of Macrophages_FABP4 is the highest, while the score of Macrophages_CXCL10 is the lowest, as shown in Supplementary Fig. S4d.

### Pathway Enrichment and Cellular Interaction Mechanism Analysis Between RUNX3-Positive and Negative Groups

Through multidimensional analyses of the RUNX3_Pos and RUNX3_Neg groups, the differences in gene expression, functional pathways, and potential biological behaviors between the two groups were systematically analyzed.

**Fig. 8:**
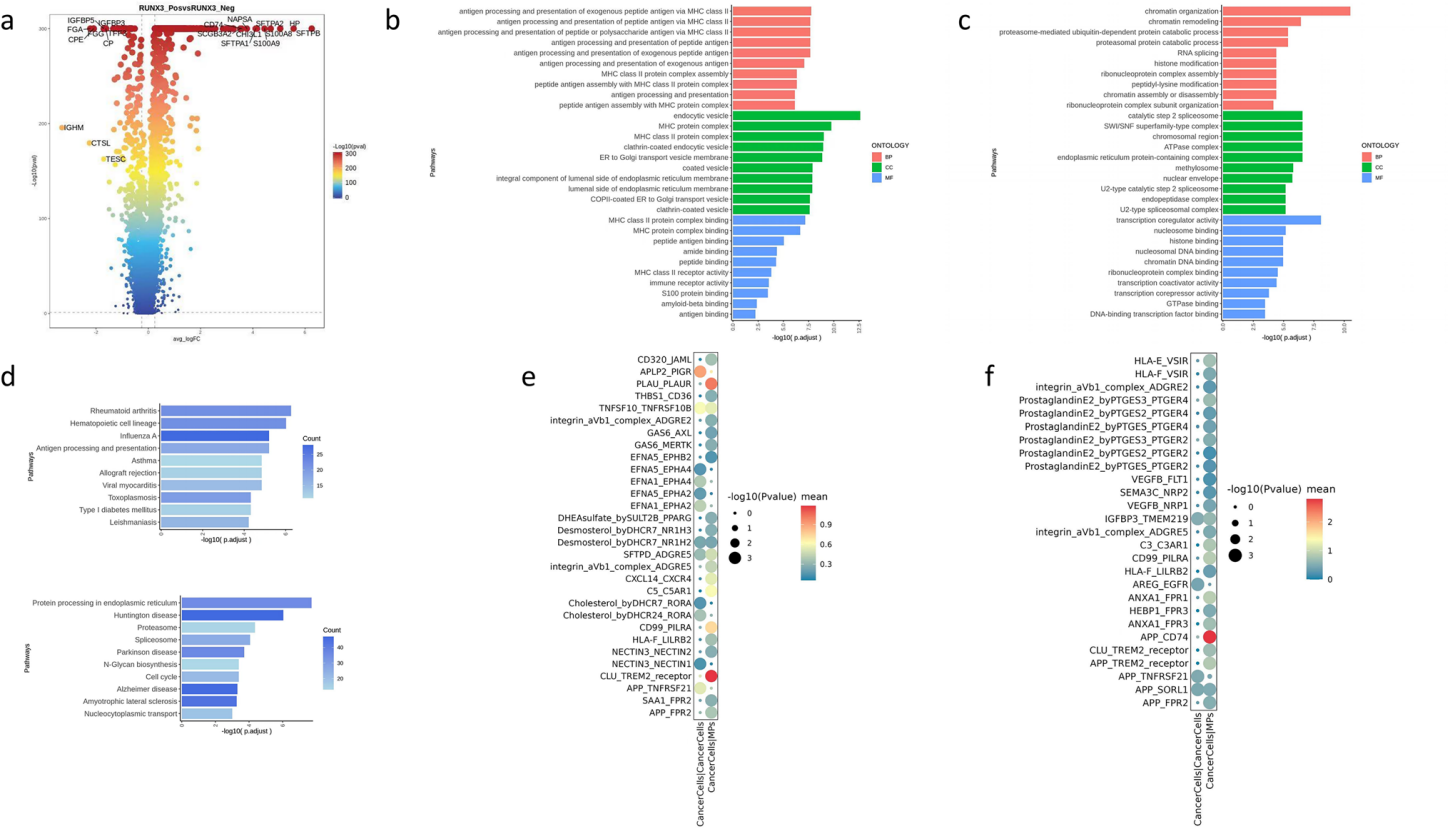
Multiomics characteristics and immune interaction analysis of RUNX3_Pos and RUNX3_Neg Cells; **a** volcano plot of differentially expressed genes between RUNX3_Pos and RUNX3_Neg Groups; **b** GO enrichment analysis of differentially expressed genes in RUNX3_Pos group (immune-related pathways); **c** GO enrichment analysis of differentially expressed genes in RUNX3_Neg group (nuclear gene regulatory pathways); **d** kegg pathway enrichment analysis, with the upper panel for RUNX3_Pos and the lower panel for RUNX3_Neg; **e** ligand–receptor interaction analysis between RUNX3_Pos tumor cells and MPs; **f** ligand–receptor interaction analysis between RUNX3_Neg tumor cells and MPs

The results of differential gene expression analysis were presented as a volcano plot (Fig. 8a). There was a large number of differentially expressed genes between the RUNX3_Pos and RUNX3_Neg groups, including S100A8, S100A9, CHI3L1, CD74, HLA-DRA/DRB1/DPA1, and so on. (Supplementary Table 10). These genes were distributed in different quadrants of the volcano plot, suggesting significant differences in the gene expression profiles of the two groups.

In the GO enrichment analysis, the differentially expressed genes in the RUNX3_Pos group were significantly enriched in immune-related pathways as shown in Fig. 8b. At the biological process (BP) level, they were concentrated in immune recognition-related processes such as “antigen processing and presentation via MHC class II molecules.” At the cellular component (CC) level, they involved immune-related structures such as MHC protein complexes and endosomes, and at the molecular function (MF) level, they covered functions such as antigen binding and peptide antigen binding. This indicated that cells with high RUNX3 expression had active immune antigen processing and presentation capabilities, which were conducive to triggering immune responses. In contrast, the GO enrichment of the differentially expressed genes in the RUNX3_Neg group, as shown in Fig. 8c, focused on nuclear gene regulatory pathways, such as chromatin organization, RNA splicing, and proteasome-mediated ubiquitin-dependent protein catabolic processes. This reflected that its gene expression regulation mode was more inclined to maintain the plasticity of the cells themselves, and the functions related to immune responses were relatively silent. The KEGG pathway enrichment results showed that the differentially expressed genes in the RUNX3_Pos group were enriched in immune-related pathways such as “antigen processing and presentation,” while the RUNX3_Neg group was associated with pathways such as “protein processing in endoplasmic reticulum” and “spliceosome,” as shown in Fig. 8d. This further confirmed the functional divergence between the two groups of cells in terms of immune activity and self-regulation from the perspective of metabolic pathways.

Through ligand–receptor interaction analysis as shown in Fig. 8e–f, the signal interaction characteristics between RUNX3_Pos and RUNX3_Neg tumor cells and MPs were systematically analyzed. The receptor–ligand pairs that frequently appeared in the RUNX3_Pos group include: EFNA–EPHA/EphB series: involved in chemotaxis, cell localization, and morphological regulation, suggesting that RUNX3+ tumor cells are more likely to affect the migration and adhesion of MPs.^24^ SAA1–FPR2 and APP–FPR2 belong to ligands related to acute-phase response and inflammation.^25^ CXCL14–CXCR4 suggests a chemotactic function; RUNX3 may induce the expression of chemokines to attract MPs.^26,27^ For the RUNX3_Neg group, ligand–receptor interaction axes such as PGE2–PTGER, APP–TREM2, and HLA-F-VSIR are more prominently concentrated.

## Discussion

In summary, using scRNA-seq, we revealed the characteristics of two subtypes of NSCLC, one being RUNX3_Pos and the other being RUNX3_Neg NSCLC. One of our pivotal findings is that these two subtypes of NSCLC demonstrated significantly different cell infiltration characteristics. Compared with RUNX3_Pos cancer tissue, RUNX3_Neg cancer tissue is more likely to enrich a large number of club cells, ciliated cells, fibroblasts, plasma cells, B cells, neutrophils, mast cells, and pDCs. Furthermore, we have found that, compared with normal tissues, MPs exhibit a depleted status in both RUNX3_Pos and RUNX3_Neg cancer tissue, indicating that MPs depletion is an important microenvironmental feature in the onset of NSCLC. We further analyzed the MPs atlas of two types of cancer tissues and found that RUNX3_Neg cancer tissues are more likely to undergo a macrophage depletion, while RUNX3_Pos tissues are more likely to demonstrate a macrophage enrichment. Thus, we have preliminarily confirmed that RUNX3 plays an important role in the regulation on the TME of NSCLC.

Notably, RUNX3_Pos significantly upregulates key immunomodulatory factors, which not only participate in inflammatory cytokine release and antigen presentation but also influence directly the migration, activation, and functional states of mononuclear phagocytes (MPs) in the tumor immune microenvironment through ligand–receptor interactions. GO enrichment analysis shows that RUNX3_Pos cells are significantly enriched in processes such as “antigen processing and MHC II-dependent presentation,” “cell adhesion,” “response to external stimuli,” and “wound healing,” indicating stronger immune recognizability and enhanced capacity for immune cell interaction. Based on multiomics results, we hypothesize that RUNX3 reshapes the immune interaction potential of tumor cells through the following pathways: (1) enhancement of antigen presentation ability by upregulating MHC class II molecules (e.g., HLA-DRA/DRB1 and CD74), thereby improving tumor cell recognition by the immune system and (2) secretion of inflammatory/chemotactic factors (e.g., S100A8/A9 and CHI3L1) to drive MP migration and chemotaxis. Thus, RUNX3 broadly influences immune signal output through transcriptional regulatory pathways, potentially playing a central role in maintaining tumor–myeloid cell interactions and providing potential intervention strategies for targeting the RUNX3–MP interaction axis.

Cell–cell interaction analysis further reveals that RUNX3_Pos tumor cells actively shape the immune microenvironment through multigroup chemokines and immunoregulatory ligands interacting with MPs. By contrast, RUNX3_Neg tumor cells retain some capacity for interaction with MPs but primarily maintain an “inhibitory/tolerogenic” state. This occurs not through active induction but possibly through passive maintenance of immunosuppression via lipid-, prostaglandin-, and immune checkpoint-mediated pathways.

In this study, scRNA-seq enabled comparisons of TME at the cellular level among three tissue types, resulting in the identification of decreased MPs of RUNX3 in NSCLC. The RUNX family (RUNXs) of transcription factors, including RUNX1–3, play key regulatory roles in cellular activities such as proliferation, differentiation, and apoptosis.^28,29^ Emerging evidence suggests that RUNXs function as either tumor suppressors or oncogenes in different types of human cancer.^12,30,31^ The duality of RUNX family roles suggests that the functions of these regulatory factors are extremely complex. Unfortunately, the specific functions of the RUNX family are still poorly understood.^32^ New data suggests that RUNX3 protein plays an important role in the transforming growth factor (TGF)-β signaling pathway that is involved in tumor growth inhibition and apoptosis. Enhanced RUNX3 expression is associated with better overall survival (OS) and higher ratio of apoptosis in lung cancer.^33^ Our previous study showed that coexpressed RUNX3 with H3K27me3 defined a subpopulation with better prognosis in NSCLC, especially in their early stage.^34^ Moreover, we have found that loss of expression of RUNX3 predicts worse outcome in NSCLC.^18^ Therefore, on the basis of the currently available evidence, we believe RUNX3 is involved in cancer regulation more as a role of TSG in NSCLC, and the clinical and pathological significance of RUNX3 in NSCLC and its potential drug-target’s role have attracted increasing attention.^35^ Abnormal hypermethylation of TSGs is the most common reason underlying gene silencing in human cancers.^36^ There is accumulating evidence suggesting that hypermethylation silencing of RUNX3 is an early molecular feature in the carcinogenesis of lung cancer.^37^ A growing amount of data indicated that RUNX3 expression was found absent in ~ 33.3–50% of NSCLC, especially in lung adenocarcinoma (LUAD).^37,38^ Meanwhile, RUNX3 hypermethylation was detected in 25% of NSCLC tumors, and it was significantly more frequent in nonsmoker patients and patients with lung adenocarcinoma.^37^ These data indicated that silencing of RUNX3 plays an especially important role in the pathogenesis of NSCLC, and aberrant methylation is an important mechanism of RUNX3 disorder in NSCLC.^39^

RUNX3 is a transcription factor that plays a regulatory role in cell proliferation and development, and suppress tumor tumorigenesis and metastasis in different human cancers.^40,41^ Many studies have explained the phenomenon and potential mechanism of its tumor suppressive function, which is reflected in its ability to inhibit cancer cell proliferation after expression inhibition, as well as its inactivation in cancer cells.^31^ In recent years, with the development of transcriptomics technology, various sequencing techniques can be used to perform transcriptome sequencing on cancer tissues, thereby revealing the gene expression characteristics of different cancer subtypes at the transcriptome level.^42^ Wang’s microarray analysis has uncovered an altered transcriptional profile underlying the cell shape change and the suppression of migration and metastasis of RUNX3 in melanoma.^43^ In the current study, we used scRNA-seq to get a differential expression profile on RUNX3_Pos and RUNX3_Neg NSCLC. We have preliminarily demonstrated the existence of significantly different tumor microenvironments in the two groups of tissues. First of all, we classified and annotated all cells at a macro level, and 13 types of cells were identified. We found that only fibroblasts were enriched in RUNX3_Pos cancer tissue, while club cells, ciliated cells, fibroblasts, plasma cells, B cells, neutrophils, mast cells, and pDCs were more enriched in RUNX3_Neg cancer tissue. Our finding indicates that RUNX3_Pos lung cancer exhibits significant tumor microenvironment suppression. We speculate that the reason for this phenomenon is the lack of expression of RUNX3 protein in lung cancer tissue, which weakens the anticancer effect and instead promotes the accumulation of a large number of noncancer cells, manifesting as nonfunctional enrichment or ineffective enrichment. Cancer tissues with positive expression of RUNX3 protein have strong anticancer effects, leading to the depletion of immune cells and a decrease in the number of immune cells, which provides a scientific explanation for better prognosis of RUNX3 overexpression in our previously reported study.^34^ As shown in Fig. 4c, both types of cancer tissues demonstrated a decrease in the number of T cells and MPs, indicating that the decrease in T cells and MPs may be a manifestation of the anticancer response in human cancer. Secondly, as mentioned earlier, we noticed a significant decrease in the infiltration of MPs in cancer tissues, indicating that this is an extremely important anticancer response. As is well known, lymphocytes play a crucial role in tumor immune surveillance. More and more evidence suggests that myeloid cells also have a significant impact on the development of tumors.^44^ Tumor-associated myeloid cells (TAMCs) are heterogeneous and have significant or even opposite effects on tumor cells and TME.^45^ In addition, TAMCs play a crucial role in regulating lymphocyte behavior, leading to immune stimulatory or immunosuppressive TME, thereby inhibiting or promoting tumor development.^45,46^ The most extensively studied TAMCs include monocytes, tumor-associated macrophages (TAMs), DCs, tumor-associated neutrophils (TANs), and myeloid-derived suppressor cells (MDSCs), which have participated in pleiotropic processes, including tumor cell growth, differentiation, dissemination, metastasis, angiogenesis, TME reshaping, immune regulation, and response to cancer treatment. Understanding the role and mechanism of TAMCs in tumors will help discover new treatment methods.^47^ From Fig. 6c, we also found differential enrichment of five subtypes of MPs in two types of lung cancer tissues. Third, we further conducted in-depth analysis on macrophages and found that cells of six subtypes of macrophages were differentially enriched in two types of lung cancer tissues. We used the relevant gene sets of M1 and M2 macrophages for gene set scoring, and the results showed a significant correlation between macrophage subtypes and M1 and M2 scores. According to the function and polarity of macrophages, they can be divided into two types: classically activated macrophages (M1 type) and alternatively activated macrophages (M2 type).^48^ The classically activated M1 macrophages are believed to have proinflammatory features, while alternatively activated M2 macrophages are believed to promote tumor growth through their anti-inflammatory function.^49,50^ The high score of M2 macrophage marker genes indicates that macrophages in the tumor microenvironment migrate towards the M2 macrophage phenotype, and the number of monocyte-like macrophages decreases, while the number of M2-like macrophages increases.^51^ However, although M1 and M2 subtypes are applicable to mouse models, they cannot fully summarize the characteristics of macrophages in human cancer patients. Single-cell studies have shown that TAMS in human cancer stroma exhibit M2-like polarization, displaying the features of M2 macrophages described in mouse tumor models.^52,53^

How to change the tumor microenvironment and achieve efficient treatment of tumors through immunotherapy and targeted therapy is a hot topic in cancer treatment. Tumors represent complex ecosystems, including tumor cells, immune cells, cancer-related fibroblasts (CAFs), endothelial cells (ECs), parietal cells, additional tissue resident cells, and dynamic, vascularized extracellular matrices embedded by these cells.^54^ There may be significant differences in the composition and functional status of the tumor microenvironment related to different types of tumors, intrinsic characteristics of tumors, tumor stages, and patient states. Understanding the complex interactions between the intrinsic, extracellular, and systemic mediators of tumor cells and disease progression is crucial for the rational development of effective anticancer treatments.^54^ The changes in the TME may be the result of a dual effect of external and internal factors.^54^ By altering the TME of human cancer, better outcomes of cancer treatment may be achieved. Several studies showed better therapeutic efficacy by promoting macrophage polarization from M2 to M1 to reverse immunosuppressive TME.^55^ Although some progress has been made in therapeutic strategies based on altering TME, TME is much more complex than we imagined, and targeting multiple targets in TME may be a future trend in reversing immune suppression. Microenvironmental heterogeneity promotes tumor evolution and is a key determinant of tumor biology, treatment response, and patient survival.^56^

Our findings preliminarily confirm the view that the expression status of the RUNX3 protein is highly correlated with the TME of NSCLC. In addition, from the perspective of subcluster analysis, the differential enrichment of MPs in different RUNX3 status was preliminarily revealed. Several limitations of our study should be mentioned. First of all, our research is based on scRNA seq, and the specific mechanisms need to be further explored in the future. Second, based on the IHS data we have collected, the overall expression intensity of RUNX3 in lung cancer tissues is relatively weak, which may be related to the characteristics of the cancer itself. Therefore, it is unknown whether RUNX3 with higher expression levels will further alter TME. In the future, we may be able to explore the impact of RUNX3 expression intensity on TME by constructing animal models. Third, clinical practice targeting programmed cell death protein 1 (PD-1)/programmed death ligand 1 (PD-L1) has shown that TME heterogeneity is a core factor affecting the efficacy of tumor immunotherapy. Subsequent research will further explore the expression distribution of RUNX3 in different types of TME, revealing its regulatory mechanisms in different time periods, and providing a basis for the clinical promotion and application of RUNX3 targeted antibody coupled drugs and therapeutic antibody drugs.

## Conclusions

Our study revealed through single-cell sequencing technology that the expression status of RUNX3 is highly correlated with TME of NSCLC. Our research has found that regulating MP enrichment may represent a potential therapeutic strategy, although experimental validation is currently lacking—including whether targeting MPs can reverse the phenotypic changes and functional validation in vivo. Future studies using animal models are warranted to verify the regulatory role of MPs in RUNX3-mediated TME remodeling. This study provides valuable insights into the anticancer mechanism of RUNX3.

## Supplementary Information

Below is the link to the electronic supplementary material.

Supplementary file1 (DOCX 903 KB)

Supplementary file2 (XLSX 10 KB)

Supplementary file3 (XLSX 12 KB)

Supplementary file4 (XLSX 11 KB)

Supplementary file5 (XLSX 10 KB)

Supplementary file6 (XLSX 10 KB)

Supplementary file7 (XLSX 10 KB)

Supplementary file8 (XLSX 9 KB)

Supplementary file9 (XLSX 9 KB)

Supplementary file10 (XLSX 9116 KB)

Supplementary file11 (XLSX 271 KB)
