# Supplementary material for: Single-Cell RNA Sequencing Reveals Potential Mechanism of RUNX3 Reshaping Tumor Microenvironment in Non-small-cell Lung Cancer
Source: Ann Surg Oncol. 2025 Sep 7;32(13):10224–37. doi: 10.1245/s10434-025-18034-w (PMC12589342; doi:10.1245/s10434-025-18034-w)
Supplement: Supplementary file 1 — Supplementary file1 (DOCX 903 KB) [file 10434_2025_18034_MOESM1_ESM.docx]

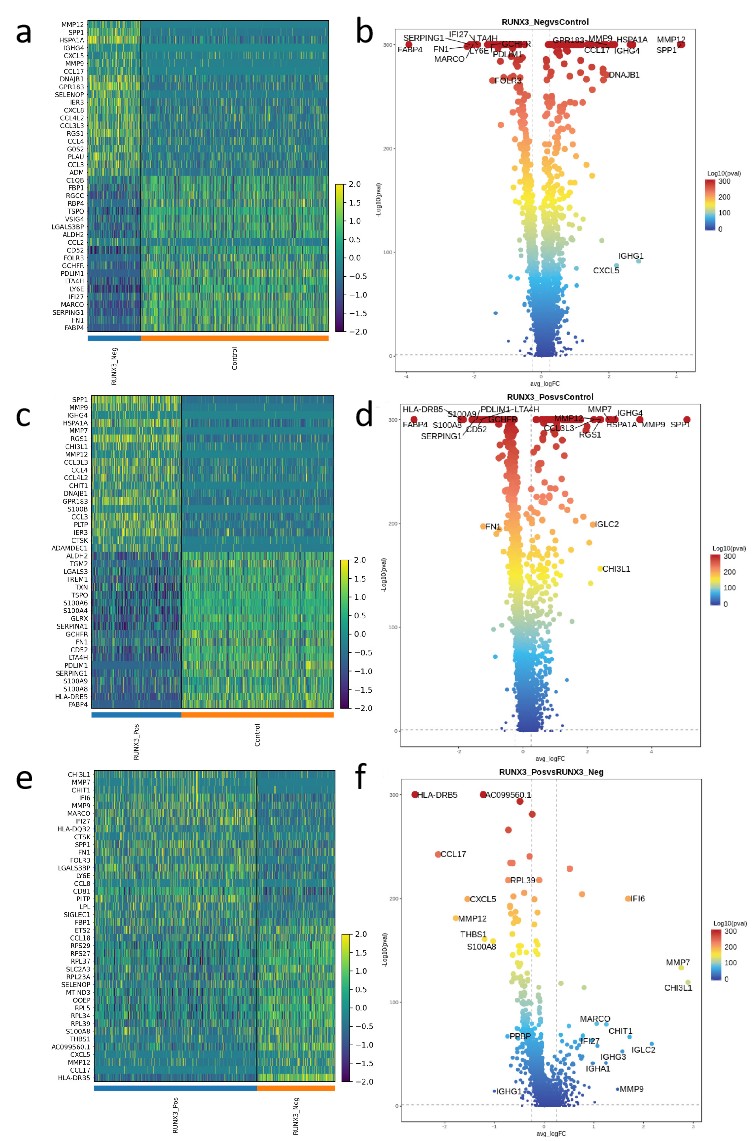


Supplementary Figure S 1 Screening of differentially expressed genes in different RUNX3 status. (a) Heatmap. The figure shows 20 genes with the most significant upregulation, 20 genes with the most significant downregulation. (b) volcano plot and the top 40 differentially expressed genes were selected to labelled. (c) Heatmap of differentially expressed genes between RUNX3 - positive and control. (d) Volcano plot of differentially expressed genes between RUNX3 - positive and control. (e) Heatmap of differentially expressed genes between RUNX3 - positive and RUNX3 - negative. (f) Volcano plot of differentially expressed genes between RUNX3 - positive and RUNX3 - negative.


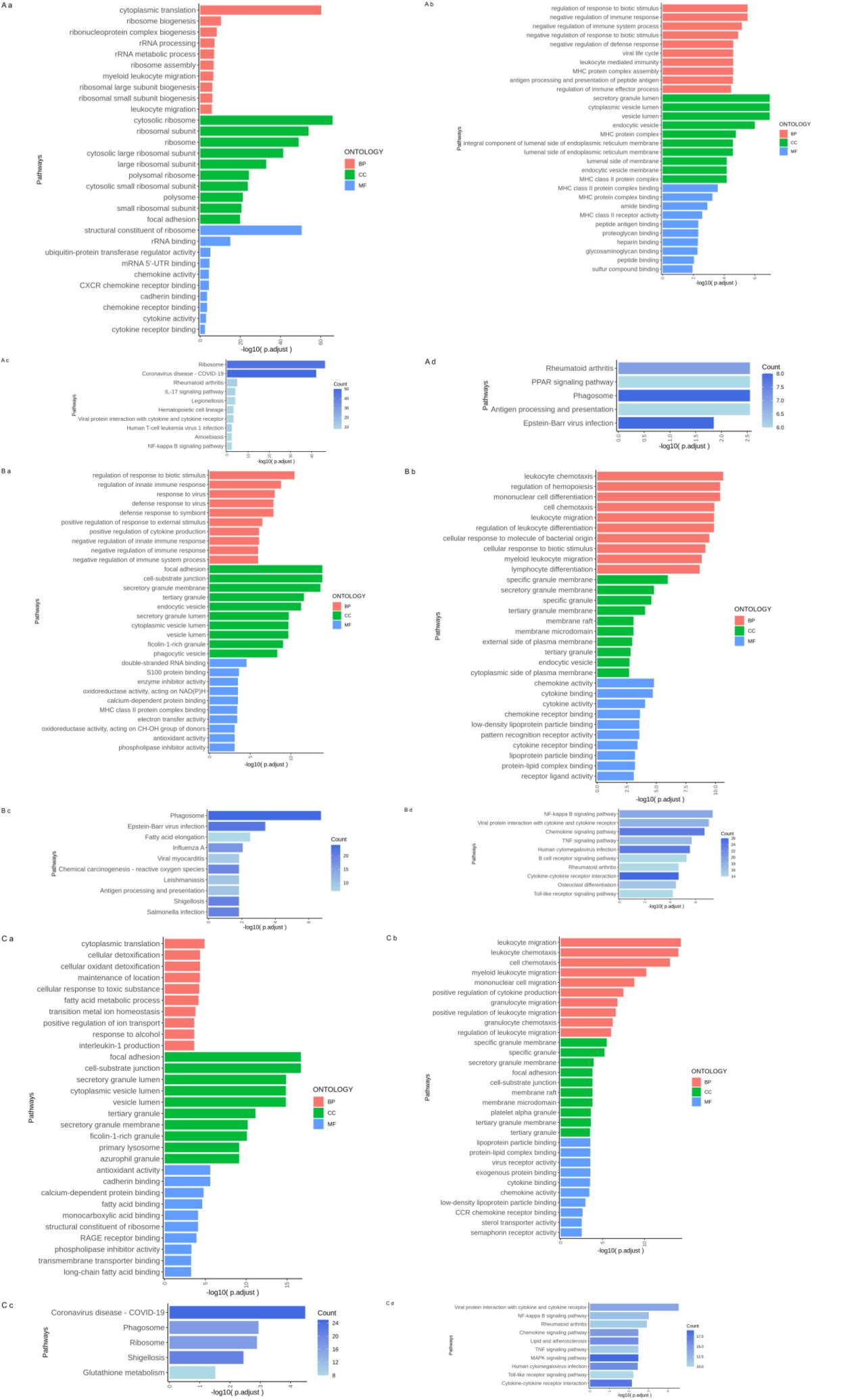


Supplementary Figure S 2 Functional annotation and pathway enrichment analysis of differentially expressed genes. (Aa) GO functional annotation of down regulated genes in control tissues. (Ab) GO functional annotation of up regulated genes in control tissues. (Ac) KEGG pathway enrichment of down regulated genes in control tissues. (Ad) KEGG pathway enrichment of up regulated genes in control tissues. (Ba) GO functional annotation of down regulated genes in RUNX3_Neg cancer tissue. (Bb) GO functional annotation of up regulated genes in RUNX3_Neg cancer tissue. (Bc) KEGG pathway enrichment of down regulated genes in RUNX3_Neg cancer tissue. (Bd) KEGG pathway enrichment of up regulated genes in RUNX3_Neg cancer tissue. (Ca) GO functional annotation of down regulated genes in RUNX3_Pos cancer tissue. (Cb) GO functional annotation of up regulated genes in RUNX3_Pos cancer tissue. (Cc) KEGG pathway enrichment of down regulated genes in RUNX3_Pos cancer tissue. (Cd) KEGG pathway enrichment of up regulated genes in RUNX3_Pos cancer tissue.


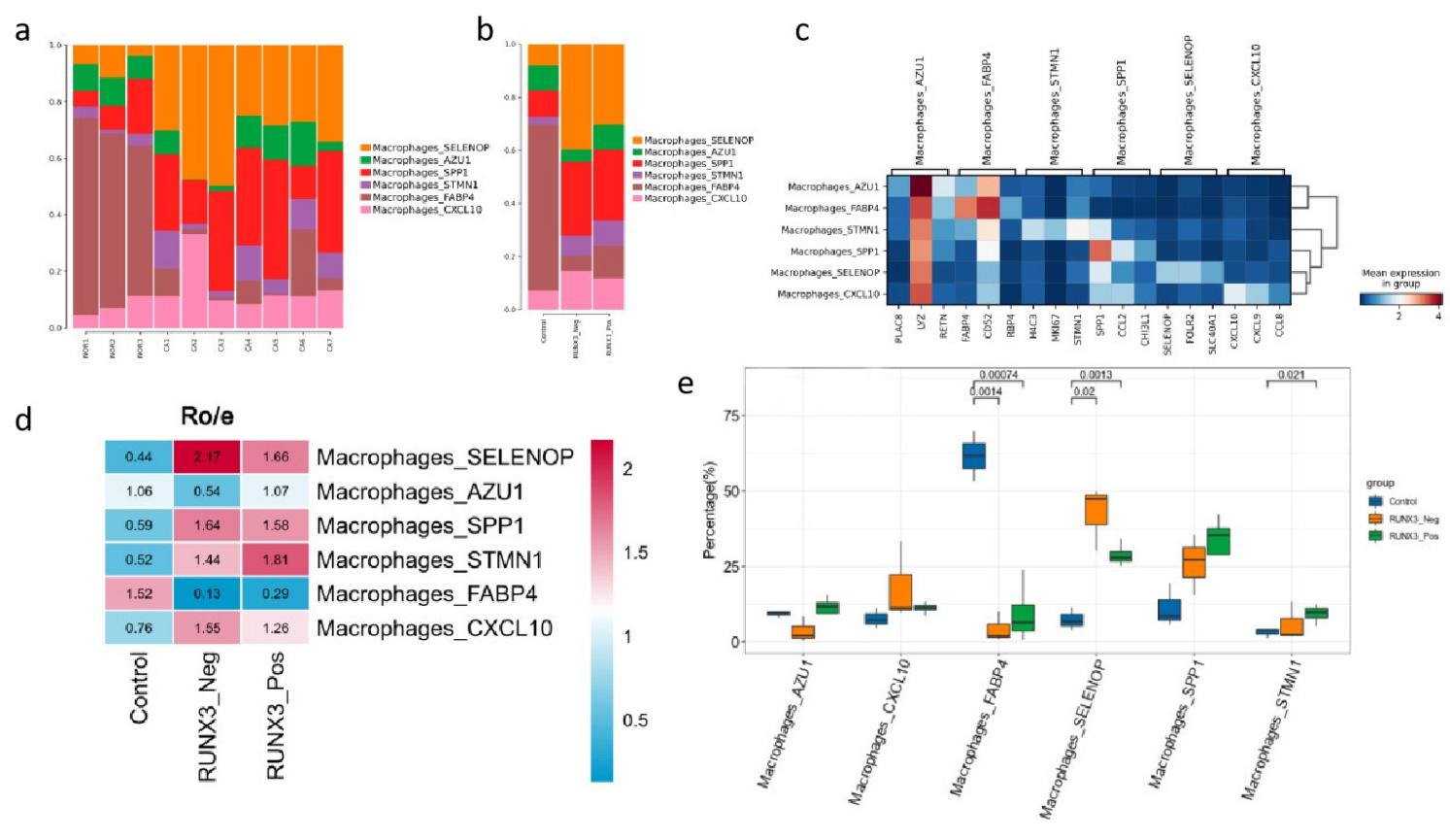


Supplementary Figure S 3 The percents of cell subclusters of mononuclear phagocytes (MPs) in each sample and group. (a) The percents of cell subclusters in each sample. (b) The percents of cell subclusters in each group. (c) Heat map of macrophage marker gene expression. (d) The results of Ro/e of cell subclusters in each sample and group. (e) Analysis of macrophage subcluster percents in different tissues.


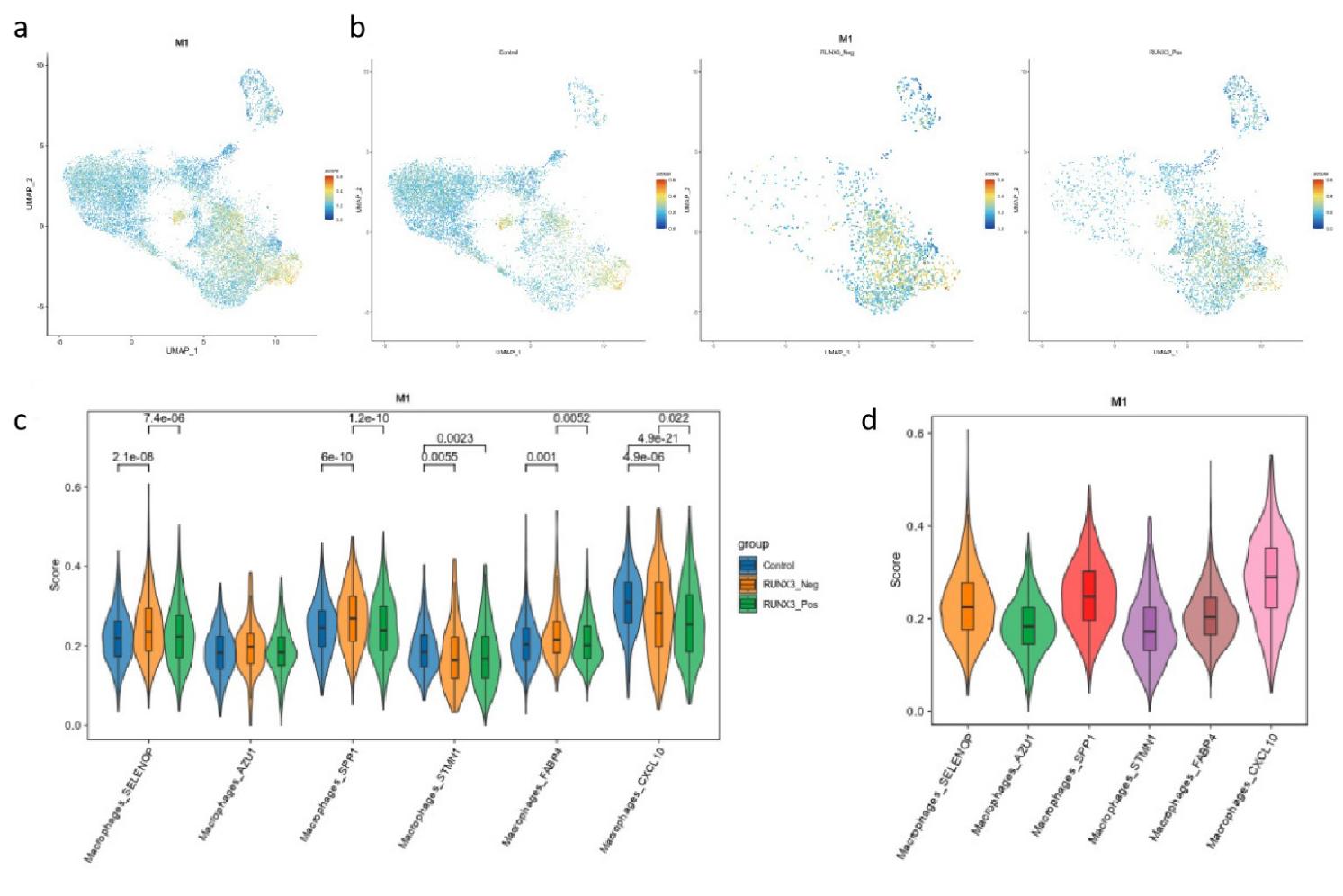


Supplementary Figure S 4 Distribution of M1 gene set scores for various macrophage subtypes in each sample and group. (a) Distribution of macrophages. (b) Distribution of macrophages grouped by histological type. (c) Heat map of macrophage marker gene expression. (d) The M1 score in each sample and subcluster.


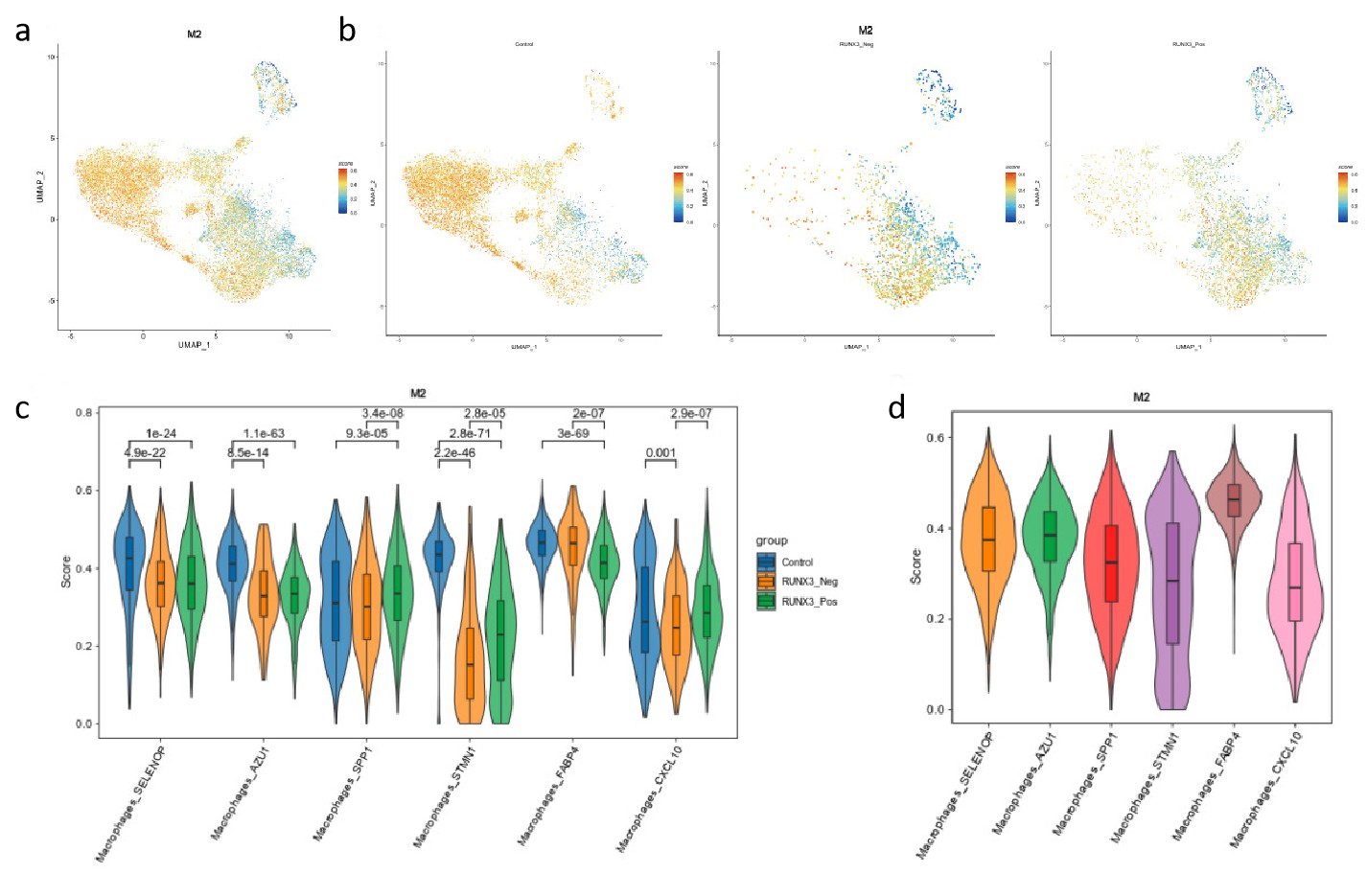


Supplementary Figure S 5 Distribution of M2 gene set scores for various macrophage subtypes in each sample and group. (a) Distribution of macrophages. (b) Distribution of macrophages grouped by histological type. (c) Heat map of macrophage marker gene expression. (d) The M1 score in each sample and subcluster.
